# Exposure to health misinformation on social media across key health domains: a systematic review and meta-analysis of survey-based studies

**DOI:** 10.1186/s12889-026-27242-2

**Published:** 2026-04-01

**Authors:** İzzet Çeleğen, Abdullah Sarıöz

**Affiliations:** Department of Public Health, Faculty of Medicine, Yuzuncu Yil University, Van, Türkiye Turkey

**Keywords:** Health misinformation, social media, Systematic review, Meta-analysis, Vaccine hesitancy, Digital literacy, Public health

## Abstract

**Background:**

Exposure to health misinformation on social media has emerged as a significant public health concern; however, survey-based evidence remains conceptually heterogeneous across health domains and outcome definitions. This systematic review and meta-analysis synthesized individual-level observational studies examining direct exposure to health misinformation across key domains, including COVID-19, vaccination, cancer, and oral health.

**Methods:**

Following PRISMA 2020 and MOOSE guidelines, PubMed, Web of Science, and Scopus were searched (2010–2025). Eligible studies were observational and survey-based, reporting prevalence or determinants of individual-level exposure to health misinformation encountered on social media. Exposure was operationalized as (i) perceived exposure (self-reported encountering of misinformation) or (ii) item-level encounter/recognition of predefined misinformation claims framed as prior exposure. Constructs reflecting belief, attitudinal agreement, susceptibility, or behavioral responses were excluded from prevalence pooling to prevent conceptual conflation. Interventional or experimental correction studies were excluded to preserve comparability with naturalistic observational exposure measures. Random-effects meta-analyses were conducted in R (meta/metafor), with heterogeneity quantified using I². Subgroup analyses were conducted by health domain, geographic region, and platform context.

**Results:**

Eight studies (*N* = 22,780) met inclusion criteria. Reported prevalence estimates varied substantially (10%–87%) across domains and exposure operationalizations. The pooled prevalence was 59.0% (95% CI: 44.0–73.0); however, heterogeneity was extreme (I² = 99.8%). Accordingly, the pooled estimate is interpreted as a descriptive contextual summary rather than a generalizable population parameter. Subgroup analyses suggested domain-dependent patterns, with comparatively higher reported exposure in COVID-19 and oral health contexts and lower levels in cancer-related contexts. Across studies, younger age, lower health or digital literacy, and minority ethnicity were recurrently associated with higher reported exposure. Platform-related associations were context-dependent and varied by outcome construct and health domain, indicating that platform effects operate as environmental modifiers rather than intrinsic determinants.

**Conclusions:**

Exposure to health misinformation on social media appears common but highly variable across health domains, operational definitions, and platform environments. Given extreme between-study heterogeneity, reliance on cross-sectional self-reported measures, and variability in exposure operationalization, findings should be interpreted as contextual rather than universally generalizable. The most informative insights derive from domain- and context-specific patterns, which may inform targeted and evidence-sensitive public health strategies.

**Supplementary Information:**

The online version contains supplementary material available at 10.1186/s12889-026-27242-2.

## Introduction

Health misinformation—defined as health-related claims that are misleading, false, or unsupported by scientific evidence [1, 2]—has emerged as a major public health challenge in the digital information age. The rapid expansion of social media platforms has created unprecedented channels for the circulation of misleading content, particularly during global health emergencies such as the COVID-19 pandemic. This phenomenon, often described as an “infodemic,” has been associated with reduced access to reliable information, weakened adherence to prevention practices, and erosion of trust in health authorities [3, 4]. Prior research indicates that emotionally salient content, cognitive biases such as confirmation bias, and algorithmic amplification mechanisms can increase the visibility and diffusion of misinformation [5–7]. These dynamics have been linked to measurable downstream outcomes, including vaccine hesitancy and treatment misconceptions [8, 9].

Although the societal consequences of misinformation have been widely discussed, empirical evidence regarding exposure to health misinformation remains conceptually fragmented. Exposure is analytically distinct from belief, endorsement, susceptibility, or behavioral response. Some studies measure perceived exposure (e.g., self-reported frequency of encountering misleading content), whereas others operationalize exposure at the item level (e.g., recognition or reported prior encounter of predefined misinformation claims) [10]. In contrast, a separate body of literature assesses downstream constructs such as belief in misinformation, attitudinal agreement, susceptibility to misinformation, or behavior influenced by misinformation ([11–12]). Conflating these constructs risks equating the occurrence of an encounter with subsequent cognitive acceptance or behavioral impact. This lack of conceptual separation contributes to methodological heterogeneity and [13] complicates interpretation across studies.

Beyond definitional variability, reported exposure patterns may differ across health domains and platform environments. Misinformation related to COVID-19, vaccination, cancer, and oral health varies in emotional salience, public visibility, and politicization, potentially influencing how frequently individuals report encountering such content. Social media platforms also differ structurally. Closed or semi-closed messaging systems (e.g., WhatsApp) may facilitate repeated circulation within trusted networks, whereas open platforms (e.g., Twitter/X) may expose users to both misleading and corrective information. Platforms such as Facebook and Instagram differ in algorithmic curation, content format, and user demographics, which may shape exposure opportunities. Accordingly, platform-related associations are best understood as context-dependent environmental conditions rather than intrinsic properties of specific platforms.

Given these complexities, a structured synthesis is needed that clearly delineates exposure constructs and systematically maps contextual variability across domains and platforms. Observational survey-based studies capture naturalistic, individual-level patterns of reported exposure and associated characteristics. In contrast, interventional or experimental studies typically manipulate misinformation or corrective content under controlled conditions to assess intervention efficacy. Because experimentally induced exposure does not reflect organic, real-world exposure prevalence, combining such designs would introduce design-based heterogeneity incompatible with prevalence synthesis. A focused review of observational survey-based evidence is therefore methodologically warranted.

The present study systematically reviews and meta-analyzes survey-based observational studies published between 2010 and 2025 that assess individual-level exposure to health misinformation encountered on social media. By explicitly distinguishing perceived exposure from item-level encounter measures—and separating exposure from downstream constructs such as belief or behavior—this review aims to provide conceptual clarity alongside quantitative synthesis. Given anticipated heterogeneity across domains, operational definitions, sampling strategies, and platform contexts [14, 15], the pooled prevalence is interpreted as a descriptive contextual summary rather than a universal population parameter. The principal contribution of this work lies in identifying domain-specific and context-sensitive patterns that may inform targeted and evidence-informed public health responses to digital health misinformation.

## Methods

### Protocol and reporting standards

This systematic review and meta-analysis was conducted in accordance with the PRISMA 2020 statement and the MOOSE recommendations for the synthesis of observational evidence [16]. A pre-specified methodological framework guided study identification, screening, eligibility assessment, data extraction, quality appraisal, and statistical analysis. The completed PRISMA 2020 checklist is provided as Supplementary File 1.

### Definition and Scope

For this review, exposure to health misinformation was defined as an individual-level encounter with misleading, inaccurate, or unsupported health information on social media platforms. Conceptual precision was prioritized to prevent conflation with downstream cognitive or behavioral constructs.

Because prior studies operationalize exposure differently, outcomes were classified into three mutually exclusive categories according to an a priori decision rule:

1. Perceived exposure.

Self-reported frequency, likelihood, or acknowledgment of encountering health misinformation on social media.

2. Item-level encounter/recognition (exposure-framed measures).

Reported prior encounter or recognition of at least one predefined misinformation claim presented within a survey instrument. Item-level measures were classified as exposure only when the survey stem explicitly assessed prior exposure (e.g., “have you seen/heard this claim?”). Measures assessing belief strength, agreement, endorsement, or perceived accuracy without an explicit encounter frame were not classified as exposure.

Item-level encounter was conceptualized as an exposure proxy because it captures reported prior contact with misinformation content independent of cognitive endorsement or evaluative agreement. This distinction was applied consistently to prevent conflation between exposure and downstream belief or susceptibility constructs.

3. Conceptually distinct downstream constructs (not pooled as exposure).

Measures of belief, attitudinal agreement, susceptibility, discernment ability, behavioral intentions, or actual behaviors (e.g., vaccine uptake). These constructs reflect cognitive appraisal or behavioral response rather than the occurrence of exposure itself and were therefore excluded from prevalence pooling.

Only categories (1) and (2) were eligible for quantitative prevalence synthesis. This classification rule was applied consistently across studies to preserve conceptual coherence. Outcomes reflecting belief-only, trust-only, attitudinal, or behavioral measures without an explicit exposure component were excluded from pooled prevalence estimates. Such outcomes were extracted separately and described narratively where relevant but were not treated as exposure measures in quantitative synthesis.

Studies examining content-level dissemination metrics (e.g., virality, reach, algorithmic amplification, or network propagation) were excluded because they assess population-level visibility rather than individual-level exposure.

### Literature search strategy

A comprehensive search was conducted in PubMed, Web of Science, and Scopus for peer-reviewed articles published between January 2010 and December 2025. Search strategies combined controlled vocabulary and free-text terms related to health misinformation, social media platforms (e.g., Facebook, Twitter/X, WhatsApp, Instagram, TikTok), and survey-based measures of exposure or determinants. Reference lists of relevant reviews were screened to identify additional eligible studies.

### Eligibility criteria

Studies were eligible if they employed an observational, survey-based design (cross-sectional, cohort, or case–control), examined individual-level exposure to health misinformation encountered on social media, and reported either prevalence estimates of exposure or determinants expressed as odds ratios or adjusted odds ratios.

To ensure conceptual clarity, studies assessing belief, attitudes, susceptibility, or behavior without an explicit exposure measure were excluded from prevalence synthesis. Studies involving traditional (non-digital) media were excluded.

Interventional or experimental studies evaluating misinformation correction, inoculation, or manipulation paradigms were excluded because exposure in such designs is artificially induced under controlled conditions. Including experimentally manipulated exposure would conflate naturalistic exposure prevalence with intervention-driven exposure scenarios and introduce design-based heterogeneity incompatible with prevalence estimation. Their exclusion was therefore methodologically necessary to preserve ecological validity and statistical comparability.

No restrictions were imposed on population characteristics, geographic setting, or health domain.

### Study selection and data extraction

Two reviewers independently screened titles, abstracts, and full texts. Discrepancies were resolved through consensus.

A standardized extraction form captured study characteristics, population demographics, health domain, platform context, operational definition of exposure, outcome type (exposure versus downstream construct), and reported effect sizes with confidence intervals.

When multiple outcomes were reported within a study, a predefined hierarchical decision rule was applied. Preference was given to measures explicitly aligned with perceived exposure or item-level encounter/recognition. If both exposure-aligned and downstream constructs were reported, only the exposure-aligned measure was eligible for prevalence pooling. Downstream constructs were extracted separately and were not treated as exposure measures in quantitative synthesis.

### Quality assessment

Methodological quality was evaluated using the Joanna Briggs Institute (JBI) Prevalence Checklist and the NIH Quality Assessment Tool for Observational Cohort and Cross-sectional Studies. Domains assessed included sampling strategy, measurement validity, confounding control, completeness of reporting, and risk of bias. Disagreements were resolved through discussion.

### Statistical analysis

Analyses were conducted in R version 4.3.1 using the meta and metafor packages.

Prevalence estimates of exposure (perceived or item-level encounter/recognition) were pooled using random-effects models with logit transformation (DerSimonian–Laird estimator). Between-study heterogeneity was quantified using the I² statistic.

Given anticipated conceptual and contextual heterogeneity arising from variation in health domains, exposure operationalization, sampling strategies, and platform environments, the pooled prevalence was interpreted as a descriptive contextual summary rather than a population-level parameter. The objective was not to estimate a single true prevalence but to characterize variability across contexts.

Determinants of exposure were synthesized using random-effects models (metagen and rma functions) only when derived from exposure-aligned outcomes. Effect sizes based solely on belief, attitudinal, or behavioral constructs were not pooled as exposure determinants but were described narratively where relevant. Forest plots display study-specific estimates, 95% confidence intervals, and inverse-variance weights on a logarithmic scale.

Subgroup analyses were conducted by health domain, platform context, and geographic region. Given the limited number of studies per subgroup, these analyses were interpreted cautiously as exploratory and context-sensitive.

Publication bias was explored using funnel plot visualization. Given the limited number of included studies, formal statistical tests for funnel plot asymmetry were not performed.

All graphical outputs, including the PRISMA diagram, forest plots, funnel plot, and risk-of-bias heatmap, were generated in R to ensure reproducibility and consistent formatting.

Despite strict eligibility criteria and harmonized outcome classification, substantial heterogeneity persisted (I² ≈ 100%), indicating that contextual differences across domains and platform environments exerted stronger influence than methodological alignment alone. Accordingly, subgroup and determinant analyses represent exploratory contextual assessments rather than confirmatory causal inferences.

## Results

### Study selection

**Fig. 1:**
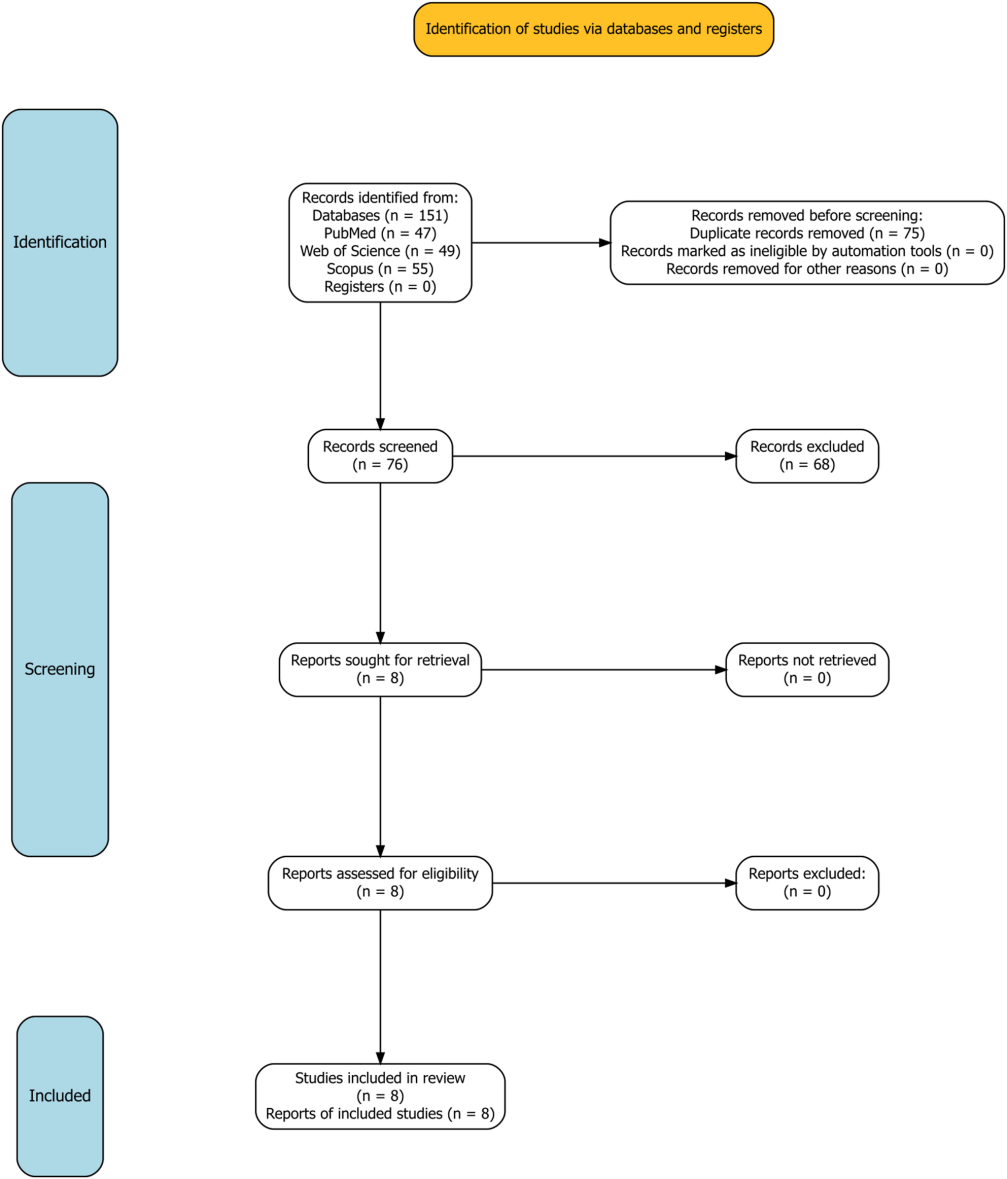
Flow diagram of the study selection process according to PRISMA 2020 guidelines

A total of 151 records were identified across the three databases searched (PubMed *n* = 47, Web of Science *n* = 49, Scopus *n* = 55). After removal of 75 duplicates, 76 unique records underwent title and abstract screening. Of these, 68 were excluded for not meeting eligibility criteria. The remaining 8 studies were assessed in full and all met predefined inclusion criteria, yielding a final sample of eight studies for qualitative synthesis and meta-analysis. The complete study selection process, including reasons for exclusion at each stage, is presented in the PRISMA 2020 flow diagram (Fig. 1).

### Characteristics of included studies

The eight included studies, published between 2021 and 2025, were conducted in geographically diverse settings [17–24]. Three were conducted in the United States, one in Spain, one in Nigeria, two in Saudi Arabia, and one in Lebanon. Five studies were conducted in high-income countries and three in lower-middle or upper-middle income contexts. All employed cross-sectional survey designs.

Sample sizes ranged from 387 to 5,041 participants and included general adult populations, university students, and caregivers of adolescent girls. Six studies relied on online convenience or panel-based sampling, while two utilized nationally representative HINTS survey data. Reporting of demographic characteristics varied across studies.

Health domains included COVID-19 misinformation (*n* = 3), vaccination-related misinformation (*n* = 2), general or cancer-related health information (*n* = 2), and oral health misinformation (*n* = 1). Platforms examined included Facebook, Twitter (X), Instagram, WhatsApp, TikTok, and Snapchat; two studies assessed social media exposure without differentiating specific platforms. Detailed study characteristics are provided in Tables 1 and 2.

**Table 1:** Characteristics of Included Studies

First Author (Year)	Country (WHO Region / Income Level)	Study Design	Population	Topic Domain	Exposure Definition	Outcome	Platform(s)
Gaysynsky (2024) [17]	USA (Americas / High)	Cross-sectional, national survey (HINTS 6)	Adults	General health misinformation	Perceived exposure; difficulty discerning misinformation	Perceived exposure & health communication behaviors	General social media
Álvarez-Gálvez (2023) [18]	Spain (Europe / High)	Cross-sectional, online survey	Adults, national panel	COVID-19 misinformation	Exposure to COVID-19 misinformation	Exposure (pooled) + Belief in misinformation (not pooled)	Facebook, Twitter, Instagram, WhatsApp, YouTube
Agha (2025) [19]	Nigeria (Africa / Lower-middle)	Cross-sectional, online recruitment	Caregivers of adolescent girls	HPV vaccination	Exposure prevalence (pooled) + vaccine uptake (secondary behavioral outcome; not pooled as exposure)	Perceived/encountered misinformation exposure (prevalence pooled); vaccine uptake analyzed as downstream outcome	Facebook, Instagram
Chandrasekaran (2024) [20]	USA (Americas / High)	Cross-sectional, national survey (HINTS 6)	Adults	General health misinformation	Exposure to misleading health information	Perceived exposure; difficulty assessing information	General social media
BinHamdan (2024) [21]	Saudi Arabia (EMRO / High)	Cross-sectional, online recruitment	Adults ≥ 15	Oral health misinformation	Item-level encounter/recognition of predefined claims (prior exposure framed)	Proportion reporting prior encounter/recognition of ≥ 1 misinformation claim (exposure)	WhatsApp, Instagram, TikTok, Snapchat, Twitter
Jabbour (2022) [22]	Lebanon (EMRO / Upper-middle)	Cross-sectional, online survey	University students	COVID-19 misinformation			Facebook, Twitter
Stimpson (2024) [23]	USA (Americas / High)	Cross-sectional survey (HINTS 6)	Adults ≥ 18	Cancer information	Perceived exposure to health mis- and disinformation on social media	Perceived exposure (included in pooled prevalence) + trust attitudes (analyzed separately)	Facebook, Twitter
Othman (2022) [24]	Saudi Arabia (EMRO / High)	Cross-sectional, online survey	Adults ≥ 18	COVID-19 vaccination	Exposure to social media vaccine information influencing decision-making	COVID-19 vaccine acceptance	Twitter, Instagram, WhatsApp

*Abbreviations*: *HINTS* Health Information National Trends Survey, *GHQ−12* 12−item General Health Questionnaire, *EMRO* Eastern Mediterranean Regional Office (WHO), *NR* Not reported. * All studies contributed one exposure−aligned prevalence estimate when an exposure−framed measure was reported. Any belief/attitude/trust/behavior outcomes reported in the same studies were extracted as downstream constructs and were not pooled as exposure prevalence

**Table 2:** Sample Characteristics of Included Studies

First Author (Year)	Sample Size (*N*)	Age (Mean/Range)	Female (%)	Sample Frame	Data Collection Year	Setting
Gaysynsky (2024) [17]	5041	NR (Adults)	~ 50% (weighted; NR explicitly)	Nationally representative (HINTS 6)	2022	National survey
Álvarez-Gálvez (2023) [18]	2200	Mean = 43.7 years	51%	National online panel	2021	Online survey
Agha (2025) [19]	4830	Caregivers of girls 9–17 (NR)	NR	Targeted recruitment via Facebook/Instagram ads (convenience)	2024	Online recruitment
Chandrasekaran (2024) [20]	5041 (analytic *n* = 4959)	NR (Adults)	NR	Probability-based national sample (HINTS 6)	2022	National survey
BinHamdan (2024) [21]	387	Adults ≥ 15 (NR)	NR	Online convenience sample (social media advertisements)	2023 (10-week period)	Online survey
Jabbour (2022) [22]	440	University students (NR)	NR	Convenience sample (Lebanese university students)	2020–2021 (COVID pandemic period)	Online survey
Stimpson (2024) [23]	4137	Adults ≥ 18	NR	National HINTS survey	2022	Cross-sectional survey
Othman (2022) [24]	504	Adults ≥ 18	NR	Snowball sampling, online recruitment	2021	Online survey

*Abbreviations*: *NR* Not reported, *HINTS* Health Information National Trends Survey

### Prevalence of exposure to health misinformation

One exposure-aligned prevalence estimate per eligible study was included in the quantitative synthesis. All included studies reported either perceived exposure or item-level exposure measures eligible for quantitative synthesis. Outcomes reflecting attitudes, susceptibility, or behavioral responses were not included in the pooled prevalence estimate.

Reported prevalence varied substantially across domains and operational definitions. The lowest estimate (10%) was observed in a cancer-related HINTS-based study (Stimpson 2024), which reported perceived exposure to health mis- and disinformation on social media. In contrast, the highest estimate (87%) was observed in an oral health context (BinHamdan 2024), based on item-level encounter/recognition measures framed as prior exposure. Nationally representative HINTS-based studies reported prevalence levels of approximately 35%, while Álvarez-Gálvez (2023) documented high levels of perceived exposure to COVID-19 misinformation in Spain.

**Fig. 2:**
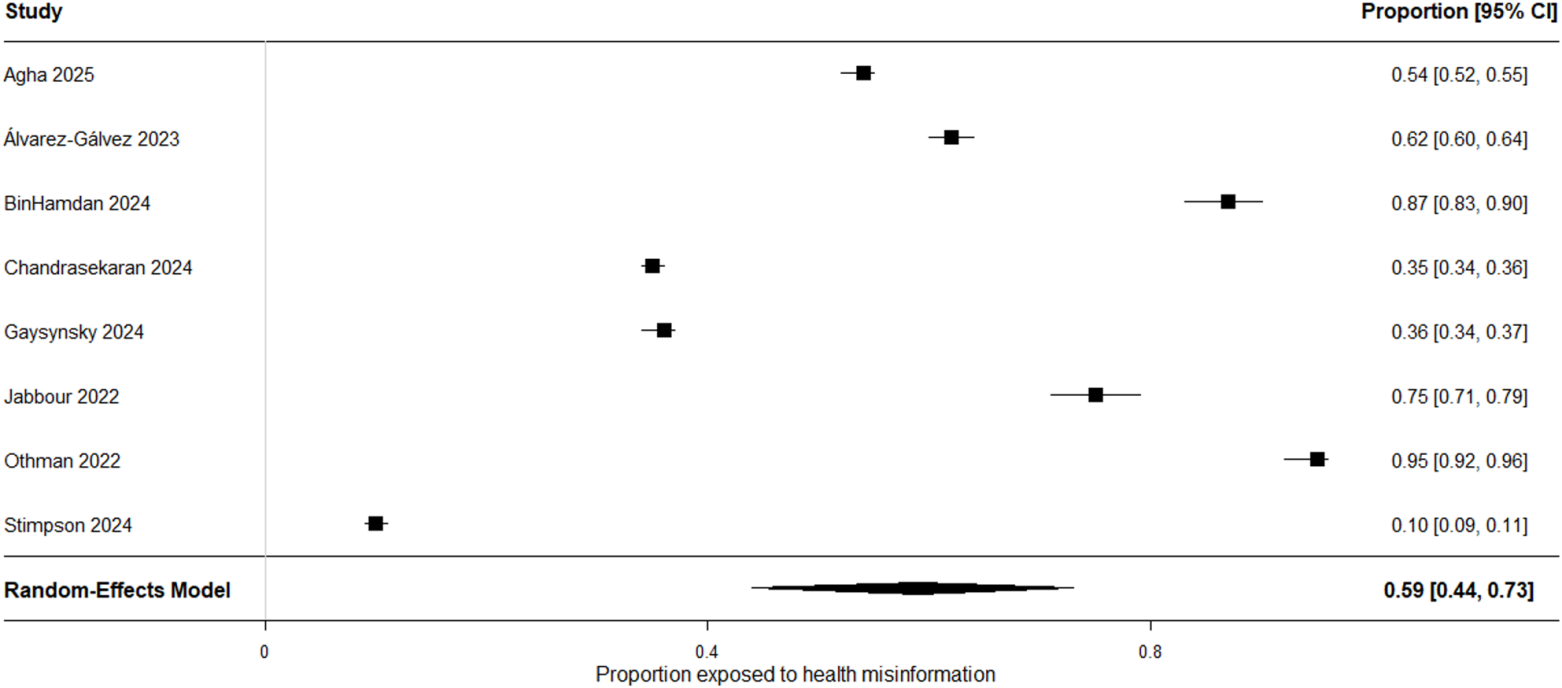
Forest plot of prevalence estimates for exposure to health misinformation across the included studies. Individual study estimates and 95% confidence intervals are shown, along with the pooled prevalence calculated using a random-effects model with logit transformation (59.0% (95% CI: 44.0–73.0)). Heterogeneity was extreme (I² = 99.8%), indicating substantial variability across health domains, measurement definitions, and sampling strategies

Using a random-effects model with logit transformation, the pooled prevalence of exposure was 59.0% (95% CI: 44.0–73.0). Between-study heterogeneity was extreme (I² = 99.8%). Accordingly, this pooled value is interpreted as a descriptive contextual summary rather than a generalizable population-level parameter. The observed heterogeneity reflects variation in health domains, exposure operationalizations, sampling strategies, and platform environments. Subgroup and sensitivity analyses were therefore prioritized to aid contextual interpretation (Fig. 2).

### Determinants of exposure

Determinants were examined across studies using socio-demographic characteristics (age, ethnicity, education, income), health or digital literacy measures, and platform-related behaviors. Meta-analytic pooling of determinants was restricted to estimates derived from exposure-aligned outcomes. Effect sizes based solely on belief, attitudinal, or behavioral constructs were not pooled as exposure determinants and are described narratively where relevant.

At the individual-study level, consistent patterns emerged. In HINTS-based analyses, Hispanic and Black respondents had higher odds of reporting exposure compared with White respondents (aOR = 1.68; 95% CI: 1.48–1.98). Lower health or digital literacy was associated with higher reported exposure (aOR = 1.87; 95% CI: 1.48–2.37). Younger adults (18–34 years) demonstrated higher reported exposure relative to older adults (≥ 65 years; aOR = 0.48; 95% CI: 0.31–0.76, reference = younger group).

**Fig. 3:**
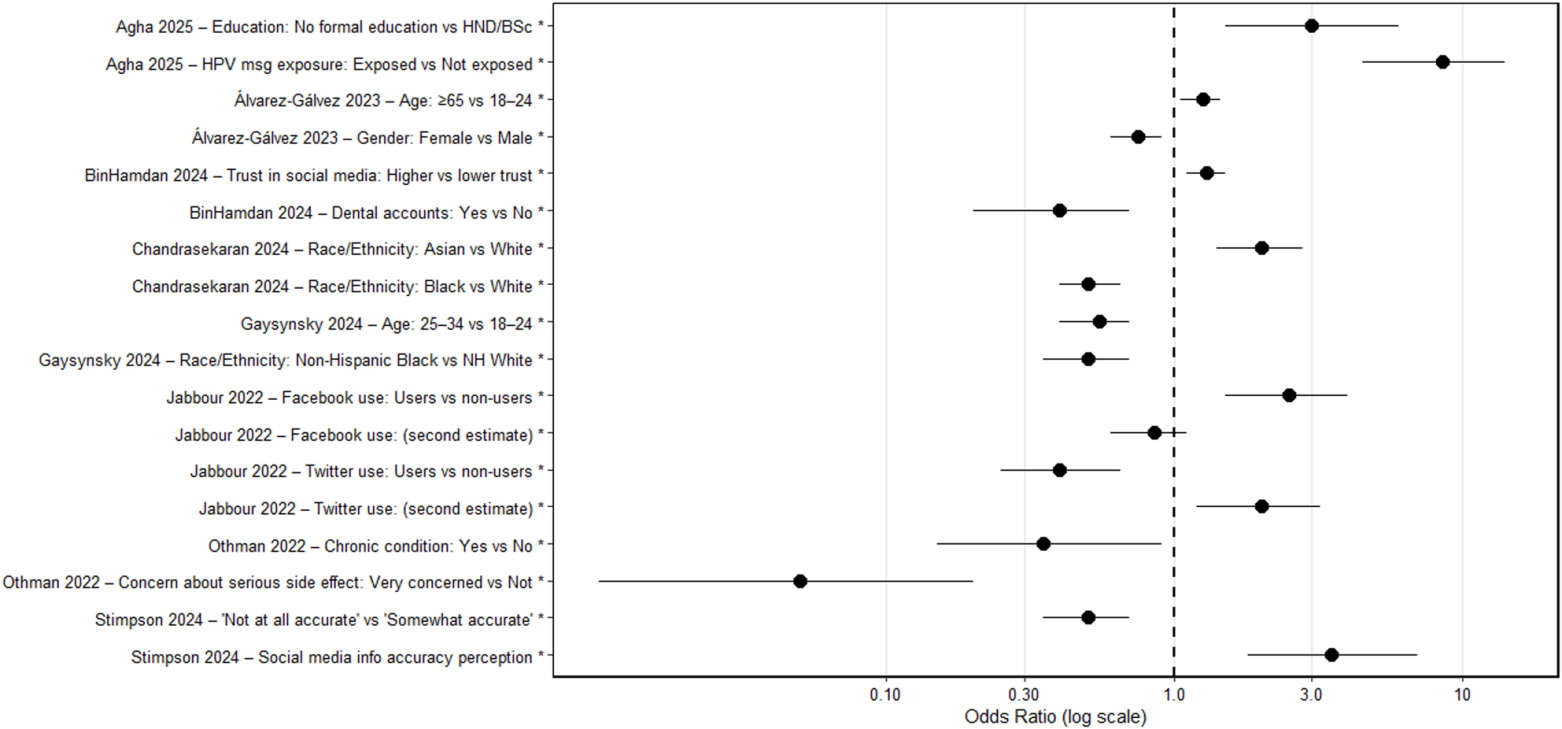
Forest plot of determinants of exposure to health misinformation across included studies. Odds ratios (OR) are presented with 95% confidence intervals (CI) on a logarithmic scale. The vertical dashed line indicates the null value (OR = 1). Estimates were synthesized using a random-effects model (DerSimonian–Laird). The pooled effect was OR = 0.90 (95% CI: 0.75–1.08), with substantial heterogeneity (I²=97.0%)

When determinant estimates were aggregated using a random-effects model, the pooled association was not statistically significant (pooled OR = 0.90; 95% CI: 0.75–1.08), with substantial heterogeneity (I² = 97.0%). This variability reflects differences in domain context, exposure definitions, population characteristics, and analytic adjustment strategies across studies. While certain predictors—particularly younger age, lower literacy, and minority ethnicity—appeared recurrent, pooled determinant estimates should be interpreted as exploratory and context-sensitive rather than definitive (Fig. 3).

### Subgroup analysis by social media platform

**Fig. 4:**
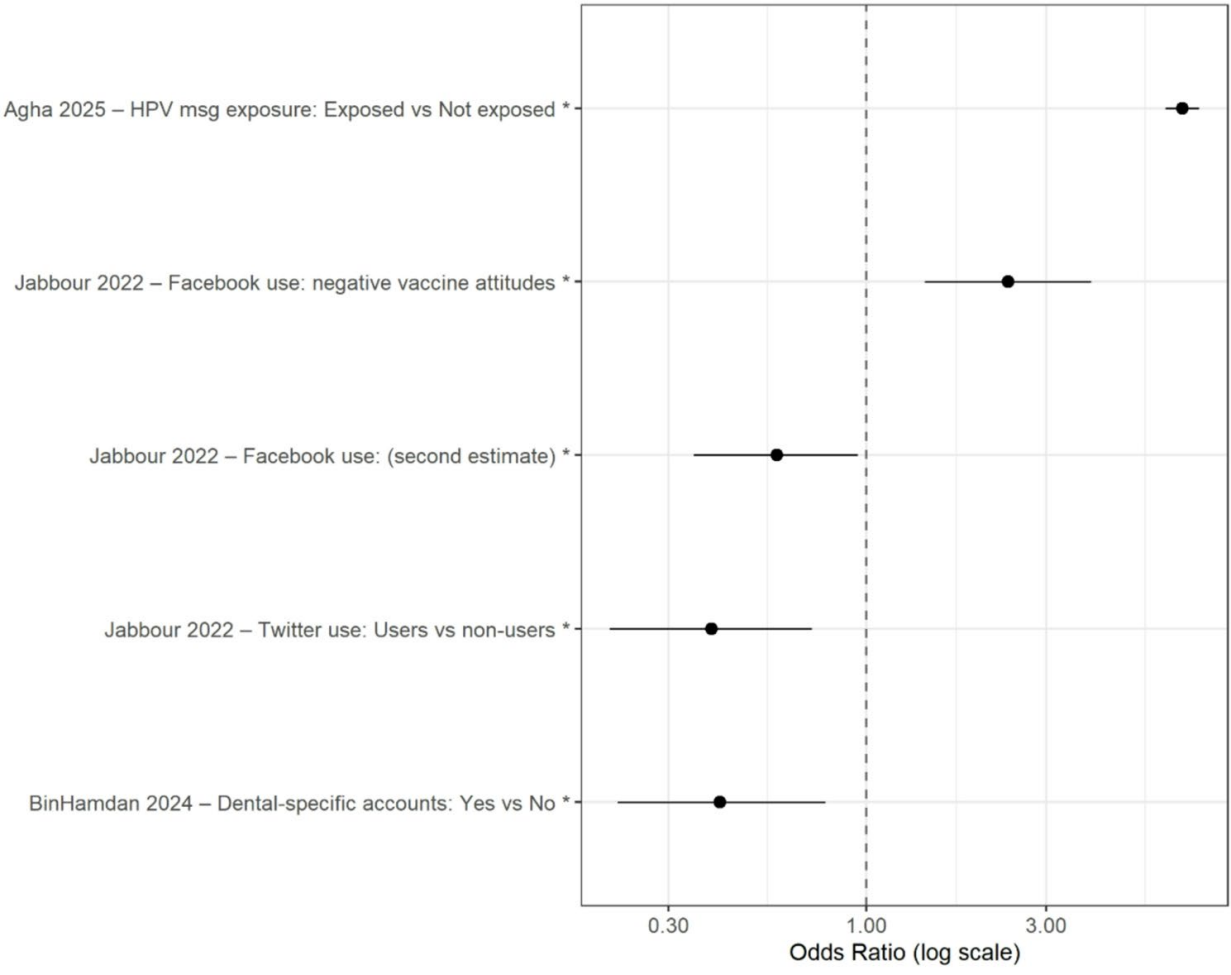
Platform-stratified associations reported across included studies. Estimates are displayed on a logarithmic scale. Outcome constructs vary across studies and include exposure measures as well as downstream attitudinal and behavioral outcomes. Accordingly, these estimates are interpreted as context-dependent associations rather than pooled causal platform effects. Platform-specific associations reflect context (content ecology, user composition, and domain-specific information environments) rather than intrinsic platform effects

Platform-related analyses demonstrated context-dependent associations (Fig. 4). Figure 4 displays study-specific platform-stratified estimates on a logarithmic scale. Outcome constructs differed across studies and included exposure measures as well as downstream attitudinal and behavioral outcomes. Because of this heterogeneity, platform-level estimates are interpreted as contextual associations rather than unified causal effects of specific platforms.

In Jabbour (2022), Facebook use was associated with higher odds of negative vaccine attitudes (OR = 2.38; 95% CI: 1.43–3.95), whereas in Agha (2025), social media exposure to HPV-related content via Facebook was associated with higher vaccine uptake (aOR = 6.87; 95% CI: 6.20–7.61). Twitter (X) use was associated with more favorable vaccine-related attitudes in one study (OR = 0.39; 95% CI: 0.21–0.72). In BinHamdan (2024), following dental-focused Instagram accounts was associated with lower levels of oral health misconceptions (aOR = 0.41; 95% CI: 0.22–0.78).

Because these estimates derive from heterogeneous outcome constructs and domain contexts, platform-related findings should be interpreted as environment-specific associations rather than intrinsic effects of particular platforms.

### Publication bias and sensitivity analyses

**Fig. 5:**
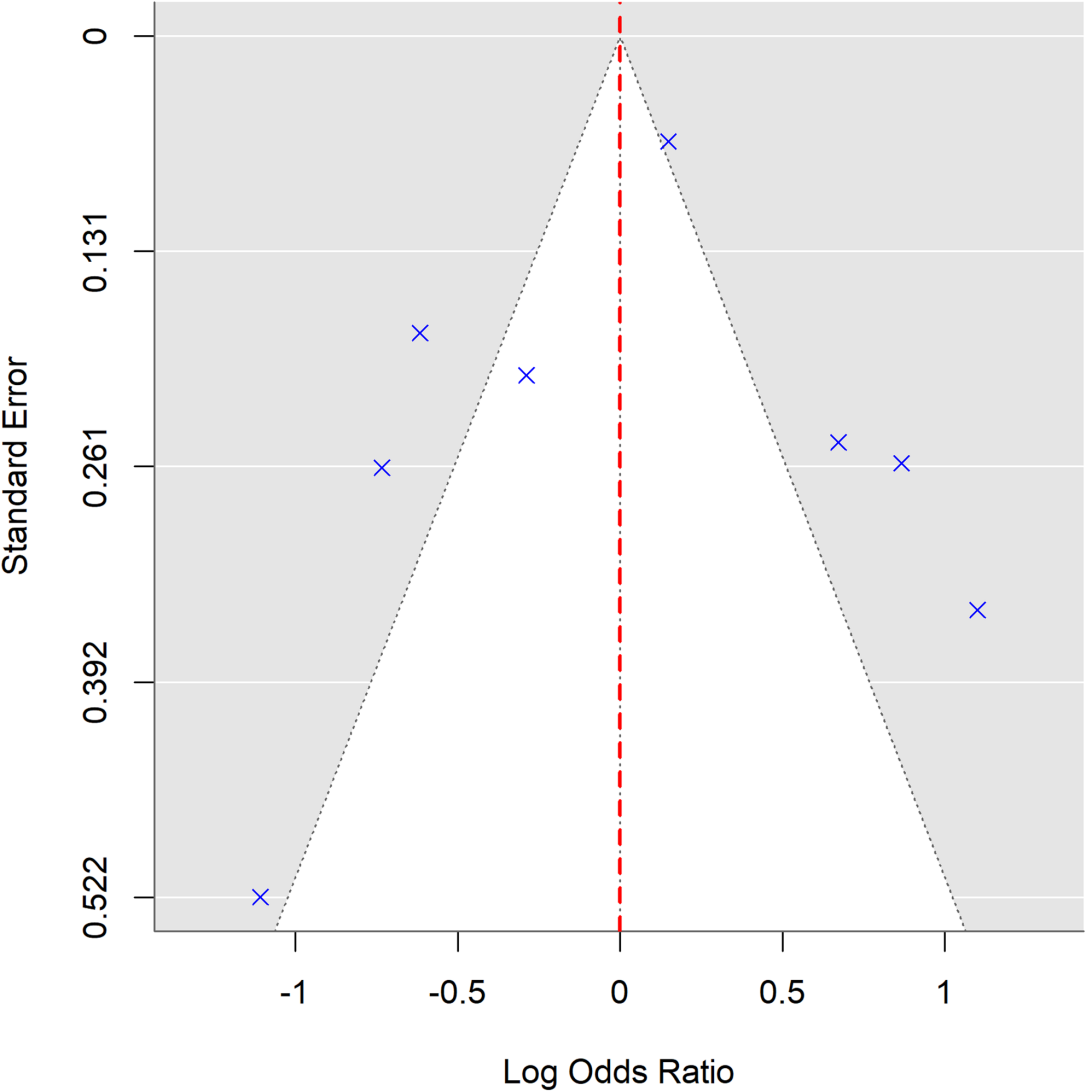
Funnel plot assessing potential publication bias. Log odds ratios are plotted against standard errors. The vertical dashed line represents the null effect (OR = 1), while the diagonal dashed lines indicate 95% confidence limits around the pooled estimate

Visual inspection of the funnel plot (Fig. 5) did not suggest pronounced small-study effects. However, given the limited number of included studies (*n* = 8), funnel plot interpretation is inherently underpowered. Formal statistical tests for asymmetry were not conducted in accordance with methodological recommendations for small meta-analyses.

Sensitivity analyses excluding studies with higher risk of bias modestly reduced heterogeneity (I² from 99.8% to 94.5%) without materially altering pooled estimates. Analyses restricted to COVID-19-related studies or excluding HINTS-based surveys yielded comparable patterns. These findings indicate that no single study disproportionately influenced overall estimates.

### Risk of bias assessment

Risk of bias varied across studies. Nationally representative HINTS-based analyses (Gaysynsky 2024; Chandrasekaran 2024) were rated as low risk across most domains. Studies relying on online convenience sampling (Agha 2025; BinHamdan 2024; Jabbour 2022) exhibited higher risk of selection bias and limited representativeness. Two studies (Othman 2022; Stimpson 2024) lacked sufficient methodological detail in certain domains, resulting in unclear ratings.

**Fig. 6:**
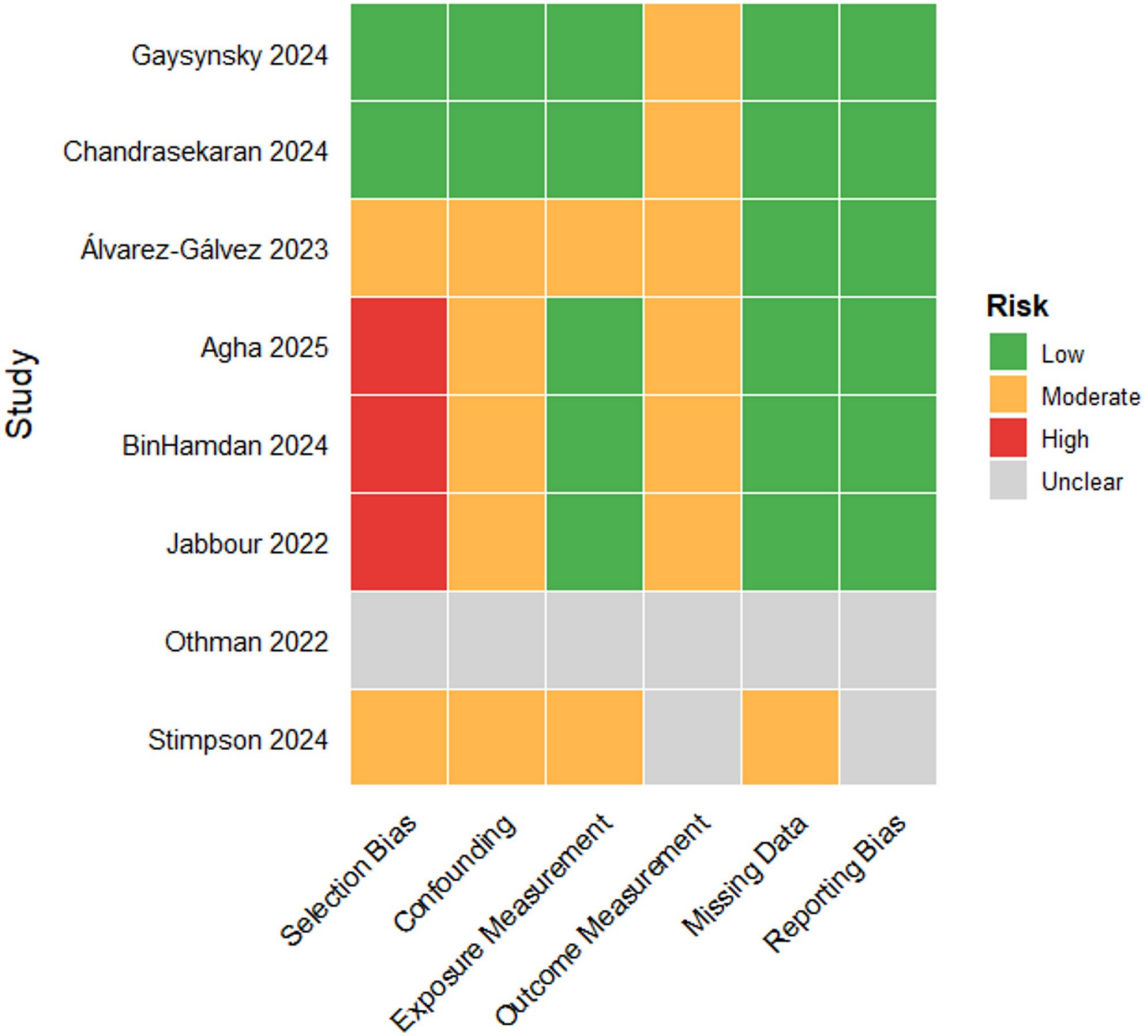
Risk of bias assessment across included studies, evaluated using the Joanna Briggs Institute (JBI) Prevalence Checklist and the NIH Quality Assessment Tool for Cross-sectional Studies. Domains included selection bias, confounding, exposure measurement, outcome measurement, missing data, and reporting bias. Judgements were classified as low (green), moderate (orange), high (red), or unclear (gray). While nationally representative HINTS-based studies (Gaysynsky 2024; Chandrasekaran 2024) demonstrated consistently low risk, studies relying on online convenience samples (e.g., Jabbour 2022, BinHamdan 2024, Agha 2025) exhibited higher risk levels

Overall, most studies demonstrated moderate risk of bias. These methodological considerations further support cautious interpretation of pooled and subgroup findings. A summary of domain-level bias assessments is presented in Fig. 6.

## Discussion

Exposure to health misinformation on social media appeared widespread across the included studies; however, the pooled prevalence estimate (59.0%) should be interpreted strictly as a descriptive contextual summary rather than a generalizable population parameter, given the exceptionally high heterogeneity observed (I² = 99.8%). Such near-total heterogeneity indicates substantial variability across contexts rather than convergence toward a single underlying prevalence. This variability reflects differences in health domains, operational definitions of exposure (perceived exposure versus item-level encounter/recognition), sampling strategies, and platform environments. These contextual differences are consistent with prior evidence demonstrating that misinformation circulates unevenly across digital ecosystems and is shaped by message characteristics, audience composition, and algorithmic amplification processes [25]. Accordingly, the pooled estimate functions as a broad indicator of magnitude within the evidence base, whereas domain- and platform-specific findings provide more interpretable contextual insights.

Prevalence estimates ranged from 10% in cancer-related contexts to over 80% in COVID-19 and oral health domains [26, 27]. This dispersion suggests that reported exposure is domain-sensitive and influenced by the salience, politicization, and emotional intensity of specific health topics. Such variability aligns with research showing that emotionally charged and identity-relevant content is more likely to circulate widely within digital environments [5, 6]. Differences in measurement framing—whether perceived exposure or item-level encounter—also contributed to variation in reported prevalence. Even within exposure-aligned constructs, item wording, recall periods, and survey context differed, reinforcing that operational heterogeneity likely amplified statistical heterogeneity. Therefore, while the pooled prevalence provides an aggregate summary of exposure magnitude, it should be interpreted within its methodological and domain-specific context.

Determinant analyses revealed patterned differences in reported exposure across socio-demographic groups. Younger adults and individuals with lower health or digital literacy consistently demonstrated higher reported exposure across studies. U.S.-based analyses further showed that racial and ethnic minority respondents were more likely to report exposure than White respondents [28, 29]. These findings are consistent with broader evidence linking structural inequalities, differential access to credible information sources, and patterns of digital engagement to uneven information environments [28, 29]. Moreover, higher trust in social media as an information source was associated with higher reported exposure in several datasets [15, 30]. These patterns should not be interpreted as causal pathways but as recurring associations observed within cross-sectional survey data.

It is important to reiterate that susceptibility, belief, and attitudinal agreement are conceptually distinct from exposure. Although exposure-aligned measures were prioritized in pooling procedures, residual variability in operational definitions across primary studies may have contributed to heterogeneity in determinant analyses. Consequently, determinant findings are best interpreted as context-sensitive associations rather than precise causal estimates.

Platform-related findings were highly context-dependent. In some studies, Facebook use was associated with higher odds of negative vaccine-related attitudes [31–33], whereas in other contexts, social media exposure to specific health content via Facebook was associated with increased vaccine uptake [34]. Twitter (X) and Instagram demonstrated associations that varied depending on whether outcomes reflected exposure measures or downstream attitudinal and behavioral constructs. These mixed patterns align with evidence suggesting that platform affordances, algorithmic curation, network homophily, and content ecology shape how information is encountered [25, 31–33]. Platform-related associations should therefore not be interpreted as intrinsic properties of particular platforms but as environment-specific patterns contingent on content type, user composition, and domain context.

When considered alongside the broader intervention literature, these findings underscore the complexity of misinformation mitigation. Evidence indicates that corrective strategies are influenced by message timing, framing, repetition, and source credibility [35]. Emerging work highlights the potential of in-feed corrections, low-friction verification prompts, and integration of authoritative health sources within digital streams [36, 37]. Conceptual syntheses further emphasize that susceptibility to misinformation is shaped by cognitive biases, affective responses, and social validation processes [38]. However, because the present review focused exclusively on observational exposure studies rather than interventional designs, conclusions regarding the effectiveness of corrective strategies remain indirect.

This review contributes to the literature in three principal ways. First, it synthesizes survey-based exposure data across multiple health domains and platform contexts while preserving conceptual distinctions between exposure and downstream constructs. Second, it identifies recurrent socio-demographic and literacy-related patterns associated with reported exposure. Third, it systematically characterizes the contextual variability underlying extreme heterogeneity in pooled estimates.

Several limitations warrant emphasis. All included studies employed cross-sectional designs, limiting causal inference. Exposure was measured through self-report, introducing potential recall and social desirability bias. Conceptual heterogeneity in exposure operationalization, even within predefined categories, likely contributed to statistical heterogeneity. Many studies relied on convenience sampling, limiting representativeness. The review did not assess algorithmic amplification using proprietary platform-level data. Restriction to English-language publications may have introduced language bias. Finally, the small number of included studies (*n* = 8) limited statistical power for subgroup analyses and publication bias assessment. These constraints reinforce the need for cautious interpretation.

## Conclusion

Exposure to health misinformation on social media appears common but highly variable across health domains, population groups, and platform environments. Younger adults, individuals with lower health or digital literacy, and racial or ethnic minority populations demonstrated higher reported exposure in several contexts. However, given extreme between-study heterogeneity (I² = 99.8%), reliance on cross-sectional self-reported measures, and variability in exposure operationalization, pooled estimates should be interpreted as contextual summaries rather than definitive population parameters.

Future research should prioritize standardized exposure definitions, longitudinal designs, and improved representativeness to reduce heterogeneity and clarify causal pathways. Addressing misinformation exposure effectively will require context-sensitive strategies that integrate evidence-based communication design, digital literacy support, and collaboration between public health institutions and platform stakeholders.

## Supplementary Information

Supplementary Material 1

## Data Availability

All data generated or analyzed during this study are included in this published article and its supplementary information files. Extracted datasets and analysis sheets are available from the corresponding author upon reasonable request.

## Abbreviations

aOR: Adjusted Odds Ratio
CI: Confidence Interval
COVID-19: Coronavirus Disease 2019
EMRO: Eastern Mediterranean Regional Office (World Health Organization)
GHQ-12: 12-item General Health Questionnaire
HINTS: Health Information National Trends Survey
HPV: Human Papillomavirus
I²: Inconsistency Index (heterogeneity measure in meta-analysis)
JBI: Joanna Briggs Institute
MOOSE: Meta-analysis of Observational Studies in Epidemiology
NIH: National Institutes of Health
OR: Odds Ratio
PRISMA: Preferred Reporting Items for Systematic Reviews and Meta-Analyses
WHO: World Health Organization
